# Comparison of two COVID-19 mortality measures used during the pandemic response in England

**DOI:** 10.1093/ije/dyad116

**Published:** 2023-08-23

**Authors:** Giulia Seghezzo, Hester Allen, Clare Griffiths, Justine Pooley, Liam Beardsmore, Sarah Caul, Myer Glickman, Tom Clare, Gavin Dabrera, Meaghan Kall

**Affiliations:** COVID-19 Vaccines and Epidemiology Division, UK Health Security Agency, London, UK; COVID-19 Vaccines and Epidemiology Division, UK Health Security Agency, London, UK; Data Product Development Division, UK Health Security Agency, London, UK; Health Analysis and Pandemic Insight Division, Office for National Statistics, London, UK; Health Analysis and Pandemic Insight Division, Office for National Statistics, London, UK; Health Analysis and Pandemic Insight Division, Office for National Statistics, London, UK; Health Analysis and Pandemic Insight Division, Office for National Statistics, London, UK; COVID-19 Vaccines and Epidemiology Division, UK Health Security Agency, London, UK; COVID-19 Vaccines and Epidemiology Division, UK Health Security Agency, London, UK; COVID-19 Vaccines and Epidemiology Division, UK Health Security Agency, London, UK

**Keywords:** SARS-CoV-2, COVID-19, mortality, mortality surveillance, COVID-19 surveillance

Monitoring COVID-19 mortality has been vital to understand the impact of the pandemic and inform the public health response. Over the course of the pandemic, a conversation has emerged about the best method for counting COVID-19 deaths and the merit of counting deaths ‘with’ COVID-19 versus ‘from’ COVID-19.

Routine mortality statistics for England and Wales are produced by the Office for National Statistics (ONS) and comprise data from death registrations, including cause of death.^1^ These statistics are published weekly and, since March 2020, have included a separate breakdown of deaths involving COVID-19. In these statistics, ONS distinguishes between deaths ‘involving’ COVID-19, where COVID-19 was mentioned as a cause of death on the death certificate, and deaths ‘due to’ COVID-19 where COVID-19 was designated as the underlying cause of death.^2^ These statistics provide a measure of deaths where clinical judgment has been used to assess whether a person’s death is related to COVID-19. For this reason, death certificates are the reference metric for cause-specific mortality. Reporting lag, caused by delays between when a death occurs and when it is registered, has presented a challenge for use during the COVID-19 pandemic. Death registrations are published with an approximate 11-day delay from when the death is registered.^3^ However, although most deaths are registered within 7 days, some deaths can take over a month to be registered (7% of deaths registered in 2020 took over a month to be registered).^4^ An ONS assessment of the quality of death certification during the pandemic noted that death registrations involving COVID-19 were of good quality.^5^

In response to the need for daily death figures to inform real-time decision making and modelling in the early days of the COVID-19 pandemic, NHS England started reporting the daily number of people who died in hospitals on 5 March 2020. On 29 April 2020, Public Health England [PHE, succeeded by the UK Health Security Agency (UKHSA) in October 2021] took over this reporting with a method that counted people who died in any setting. PHE began counting people who died following a positive SARS-CoV-2 test in England (also referred to as ‘deaths with COVID-19’).^6^ Death notifications were collated daily from multiple sources, using both active and passive reporting systems and linked to positive SARS-CoV-2 tests, to provide a comprehensive measure with an average 3-day reporting delay. At the time, ONS and UKHSA published a joint explanation of the differences between the two measures to ensure public transparency.^7^ The UKHSA measure was refined over time, and in August 2020, a 28-day interval was applied to deaths following a positive SARS-CoV-2 test to ensure the measure only reflected deaths during the acute phase of infection. In February 2022, the definition was updated to include deaths following re-infection.^8–10^ Between 1 April 2020 and 31 December 2022, a total of 177 253 deaths within 28 days of a positive specimen were reported, with a median time to death of 10 days [interquartile range (IQR) 5 to 16]. Of those captured in the 28-day measure, 70% of people died within 14 days of a positive test.

Both the 28-day death measure and death registrations were critical measures during the pandemic and were published in parallel on the GOV.UK COVID-19 Dashboard.^11^ The 28-day death measure was a near real-time proxy of COVID-19 deaths, reported on a daily basis and widely used by media, the public and decision makers, whereas death registrations followed 1–2 weeks later, providing more detailed analysis of people who died with COVID-19, including information on place of death and age-standardized mortality rates.

With two measures in the public domain, it was important to monitor how closely the rapid 28-day death measure approximates death registrations, to ensure face validity of the 28-day measure as a proxy for COVID-19 deaths. Reviewing deaths between 1 March 2020 and 31 December 2022, we present an assessment of how these two measures related during this period, comparing the deaths reported under the 28-day death measure linked to their subsequent death registration.

Between 1 March 2020 to 31 December 2022, there were a total of 183 618 death registrations that mentioned COVID-19 and 177 253 deaths recorded within 28 days of a positive test. For this analysis, 175 325 deaths could be linked to a death certificate. Of these, 82.6% (144 886/175 325) of deaths within 28 days of a positive COVID-19 test also had COVID-19 mentioned as a cause of death anywhere on their death certificate, and 78.9% (144 886/183 618) of all COVID-19 death registrations were reported under the 28-day measure. Furthermore, 70.7% (123 952/175 325) of deaths within 28 days of a positive COVID-19 test had COVID-19 listed as the underlying cause of death (Figure 1). Despite the large overlap between the two measures, there were two notable periods where the measures diverged: one during the early part of the pandemic in March and April 2020, and one later in the pandemic from January 2022 onwards (Figure 2).

**Figure 1.**
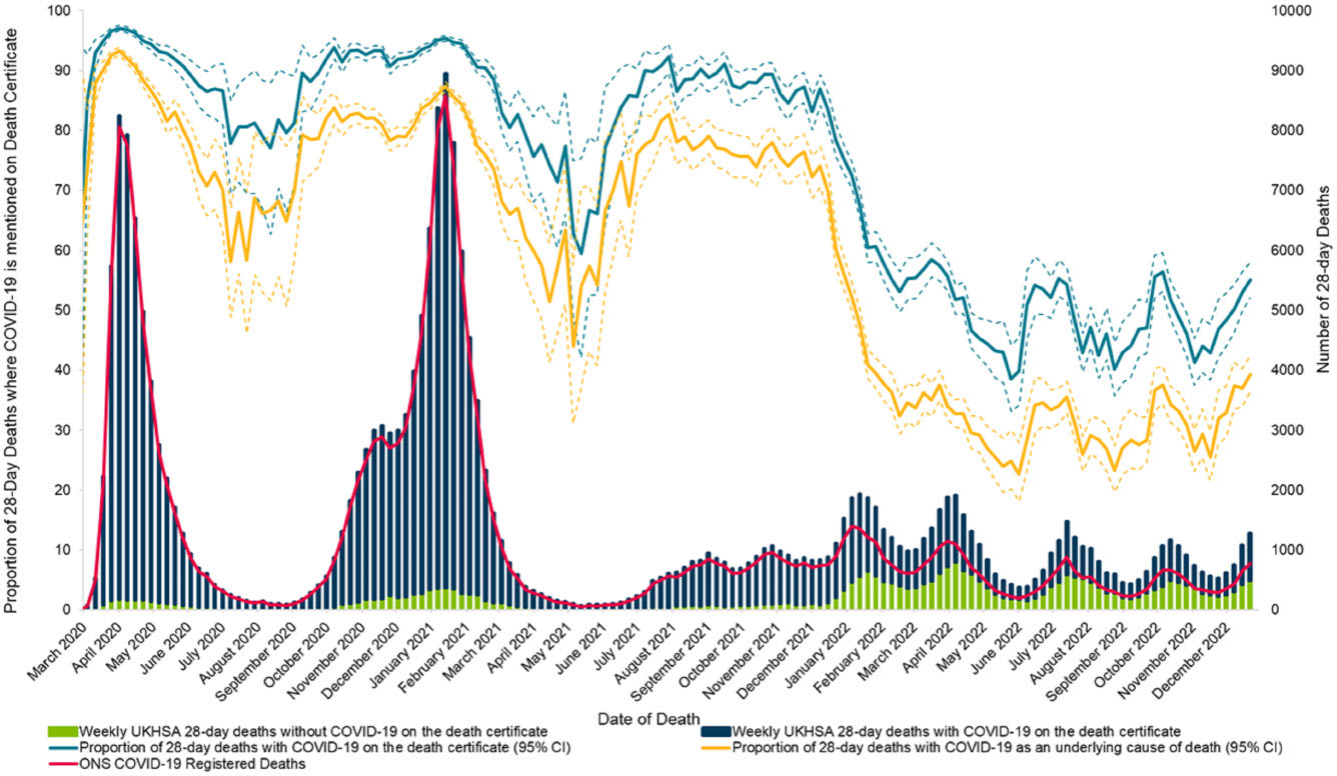
Weekly COVID-19 deaths reported by Office for National Statistics (ONS) and UK Health Security Agency (UKHSA) and proportion of 28-day deaths with COVID-19 mentioned on the death certificate, 1 March 2020 to 31 December 2022. COVID-19 death certificates are defined as where COVID-19 was mentioned anywhere on the death certificate, either as the underlying cause of death or as a contributory factor in the death, using the International Classification of Diseases 10th Revision (ICD-10) cause codes U071, U072, U99 and U109. The proportion is calculated as the weekly number of deaths reported in the 28-day measure where COVID-19 was mentioned on the death certificate, over the number of 28-day deaths where death certificate information is available [98.9% (175 325/177 253) of 28-day deaths had a death certificate available)

**Figure 2.**
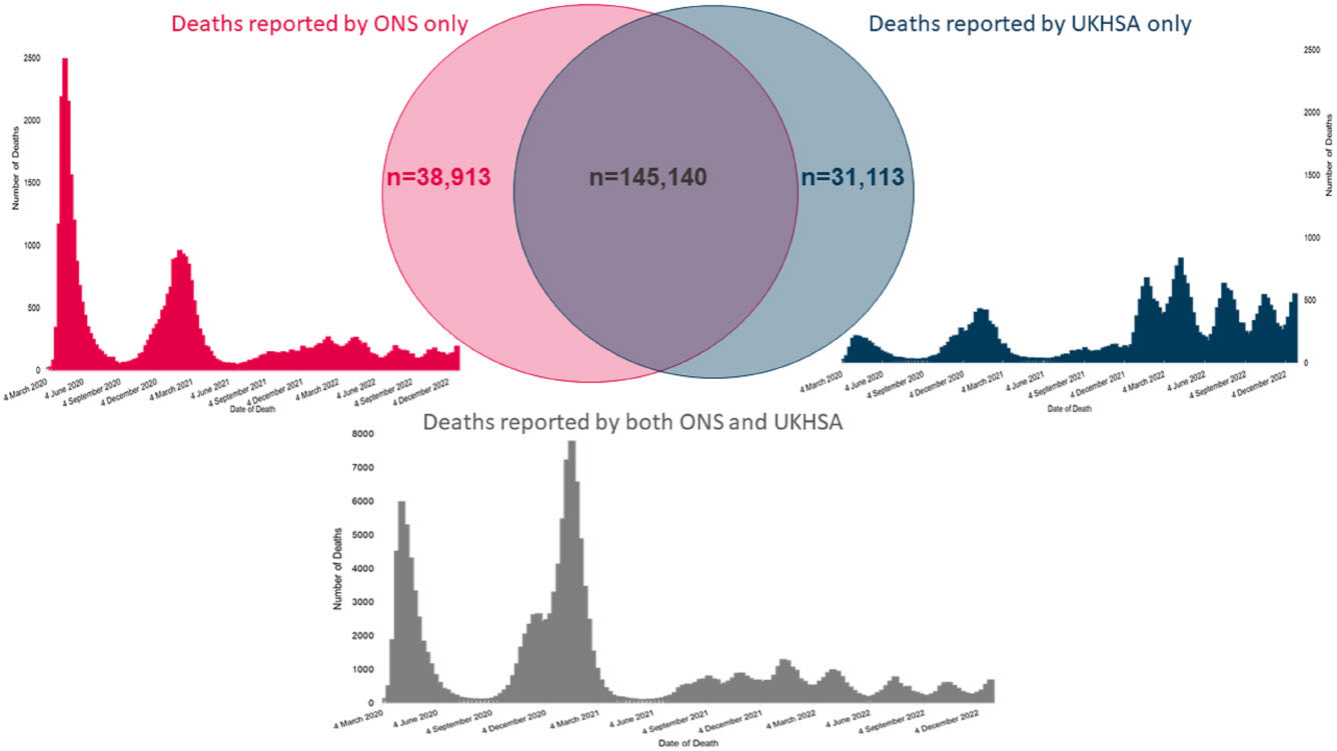
COVID-19 deaths reported by Office for National Statistics (ONS) (death certificates) and UK Health Security Agency (UKHSA) (deaths within 28 days of a positive COVID-19 test), 1 March 2020 to 31 December 2022

During March and April 2020, the number of deaths with COVID-19 mentioned on the death certificate (34 008) exceeded the number reported within 28 days of a positive SARS-CoV-2 test (25 427) (Figure 1). This was a consequence of limited testing capacity at the start of the pandemic, when many people who died from COVID-19 were not tested prior to their death, particularly in care homes and in the community (Supplementary Table S3, available as Supplementary data at *IJE* online). Following the rapid expansion of community testing after April 2020,^12^ the two measures converged, and the proportion of reported deaths within 28 days of a positive test that also had COVID-19 on the death certificate consistently exceeded 90%. The overlap between the ONS and UKHSA measures was particularly high during the Alpha and Delta waves when the number of people who died was high (Figure 1).

From January 2022, the two measures began to diverge. The proportion of people who died within 28 days of a positive test that also had COVID-19 on the death certificate dropped sharply from 80–90% to 55% by February 2022, and fluctuated between 40% and 50% through to December 2022. The proportion of deaths reported within 28 days of a positive test that had COVID-19 as the underlying cause of death dropped from 60–80% to 20–40% by December 2022 (Figure 1).

This divergence corresponded with the swift emergence of the Omicron BA.1 variant in November 2021 and dominance by late January 2022. The Omicron variant was associated with less severe illness and lower risk of death compared with previous variants.^13^ Additionally, high levels of immunity in the population acquired through vaccination and infection mediated the severity of COVID-19 disease. By the end of December 2021, 82% of the eligible English population had received two doses of the vaccine, and 85% of those aged 65 and over had received three doses. Furthermore, an estimated half of the English population were infected with COVID-19 in the 5-month period from December 2021 to April 2022, and the estimated attack rate (the fraction of the population that had ever been infected) rose from 40% to 77%.^14^ A similar divergence was observed in COVID-19 hospitalization statistics at the same time: among general admissions, the proportion of patients with COVID-19 for whom COVID-19 was the primary reason for admission declined from approximately 75% between June to mid-December 2021 to 35% by June 2022.^15–17^

We found no evidence to suggest that changes in testing policy and behaviour played a major role in this second divergence. There were no changes in testing policy when the divergence first emerged: testing was widespread, with 1–3 million polymerase chain reaction (PCR) or lateral flow tests reported daily between September 2021 and March 2022.^16^ However, subsequent changes to testing policy included ending free community testing from 1 April 2022 and stopping asymptomatic screening of hospital patients and care home residents from 31 August 2022. One might expect the two measures to reconverge when fewer asymptomatic cases were being detected, yet this was not observed. In fact, the reduction in testing provision did not lead to a reduction in the 28-day measure at all, suggesting that testing in hospital and care home settings ensures that most people who are at high risk of death from COVID-19 are diagnosed before death.

The demographic profile of the people who were reported under the two measures otherwise were very similar in terms of age, sex, index of multiple deprivation (IMD), and region of residence (Supplementary Tables, available as Supplementary data at *IJE* online). When comparing the settings of these individual’s deaths, the ONS measure included more deaths occurring in care home (20.6%) and at home (6.6%), than the UKHSA 28-day measure (15.6% and 4.9%, respectively) reflecting the limited testing outside hospital settings at the start of the pandemic (Supplementary Tables).

By 2022, over half of deaths counted in the 28-day death measure did not have COVID-19 mentioned on their death certificate. This was always a risk of defining a COVID-19 death without considering cause of death, and since the beginning of the pandemic, the 28-day measure has included some so-called background deaths, that is deaths occurring proximal to a positive test but not necessarily related to the infection. It was noted early on in the pandemic by some observers that measuring COVID-19 deaths within a set number of days following a positive test had the potential to include people who died due to causes unrelated to COVID-19.^18^ However, this remained a minority of deaths. For example, external causes of accidental injury (ICD-10 code V01-X59) accounted for a very small number (953; 0.5%) of deaths within 28 days of a positive test. Of the 17% (30 439/175 325) of deaths counted in the 28-day measure that had no mention of COVID-19 on the death certificate, the most common causes of death were health- and age-related, such as dementia, cardiovascular disease and cancer. Statistically, these deaths contributed a small proportion to the daily count of deaths when COVID-19 mortality was high during 2020 and 2021, but during periods when COVID-19 mortality was low (such as summer of 2020 and spring 2021), such deaths comprised a larger proportion of reported 28-day deaths (Figure 1).

Death registrations consider clinical judgement of the cause of death, and this may also introduce bias into how deaths are recorded. With reduced severity of disease and widespread vaccination, the contribution of COVID-19 to the events leading up to death has become less clear. This may result in subjective bias in the attribution of COVID-19 to the events leading up to death. This may be particularly true during the beginning of the pandemic for deaths in care homes and in the community which were not tested prior to death, as seen in Supplementary Table S3 . This has also been a challenge in counting deaths from other respiratory infections such as flu and pneumococcal disease, where patients present with general respiratory symptoms but there is limiting testing for these specific infections outside hospital settings.^19^ Likewise, heightened clinical awareness can influence death reporting, as was seen in 2005 when a spike in death reports involving methicillin-resistant *Staphylococcus aureus* (MRSA) was observed following increased media coverage and a directive from the Chief Medical Officer on reporting MRSA deaths.^20^ It is possible that more recently COVID-19 is less likely to be listed on the death certificate, as clinical awareness fades and the contribution of SARS-CoV-2 infection to the cause of death is more complex. Under-reporting may explain the waveform observed in the 28-day measure where COVID-19 was not listed on the certificate since January 2022 (Figure 1). Further research is needed to understand whether clinician awareness of COVID-19 influences recording on death registrations.

It remains a priority to monitor the burden of COVID-19 mortality going forward. By using consistent definitions, both of these measures remain useful to observe trends over time. The 28-day death measure remains more timely, whereas death registrations are a better estimate of the overall burden of COVID-19 mortality. A further metric to inform public health response and measure burden is excess mortality.^21^ Although subject to a reporting lag, it is a useful measure of the total impact of the pandemic, greater than COVID-19 death numbers alone, has been used to show the impact of COVID-19 when testing was low and clinical understanding was limited at the beginning of the pandemic and can be used to triangulate against the other measures.

For any emerging infection, there will be initial uncertainty about how to measure mortality associated with it. Surveillance of deaths in persons with COVID-19 was adapted to meet the demands of public health officials for timely information, resulting in the 28-day measure.

Following the end of this public health emergency of international concern, there is less need for a reporting system to provide rapid numbers and trends and we can instead focus on death registrations and excess mortality estimates. However, the experience of the pandemic has shown the usefulness of having a rapid death measure to run alongside a measure based on clinical judgement. A system to report deaths occurring within 28 days of a positive test may prove valuable in the future for COVID-19, for example in the event of a new variant with increased severity or for any future pandemic pathogen. Finally, studies are needed to better understand the contribution of COVID-19 in the sequence of events leading to death in the context of widespread immunity and a more specific population at risk of being severely affected by COVID-19, namely the elderly and immunosuppressed.^22^

## Ethics approval

Not required: authors are already able to access anonymized dataset, and it is not possible to identify individuals from the information provided.

## Supplementary Material

dyad116_Supplementary_Data

## Data Availability

The data analysed during this study are not publicly available, due to a need to protect individuals’ anonymity. These data are confidential, but fully anonymized data may be available from the corresponding author on reasonable request. Aggregated and anonymized output from the dataset described is publicly available at: [https://coronavirus.data.gov.uk/details/deaths?areaType=nation&areaName=England].
