# Supplementary material for: Comparison of two COVID-19 mortality measures used during the pandemic response in England
Source: Int J Epidemiol. 2023 Aug 23;53(1):dyad116. doi: 10.1093/ije/dyad116 (PMC10859127; doi:10.1093/ije/dyad116)
Supplement: dyad116_Supplementary_Data [file dyad116_supplementary_data.docx]

**Supplementary data**

Table S1: Distribution of COVID-19 deaths reported by UK Health Security Agency (UKHSA) and Office for National Statistics (ONS), by 10-year age groups

| Age Group | UKHSA 28-day measure | % | ONS COVID-19 death registrations | % |
| --- | --- | --- | --- | --- |
| 0-9 | 65 | 0.04% | 54 | 0.03% |
| 10-19 | 106 | 0.06% | 92 | 0.05% |
| 20-29 | 369 | 0.21% | 355 | 0.19% |
| 30-39 | 1,153 | 0.66% | 1,189 | 0.65% |
| 40-49 | 2,990 | 1.71% | 3,252 | 1.77% |
| 50-59 | 8,542 | 4.87% | 9,310 | 5.07% |
| 60-69 | 18,627 | 10.62% | 19,585 | 10.67% |
| 70-79 | 40,845 | 23.30% | 41,744 | 22.74% |
| 80+ | 102,627 | 58.54% | 108,026 | 58.84% |

Table S2: Distribution of COVID-19 deaths reported by UK Health Security Agency (UKHSA) and Office for National Statistics (ONS), by gender

| Gender | UKHSA 28-day measure | % | ONS COVID-19 death registrations | % |
| --- | --- | --- | --- | --- |
| Female | 78,758 | 45% | 82,916 | 45% |
| Male | 96,565 | 55% | 100,693 | 55% |

Table S3: Distribution of COVID-19 deaths reported by UK Health Security Agency (UKHSA) and Office for National Statistics (ONS), by place of death

| Place of Death | UKHSA 28-day measure | % | ONS COVID-19 death registrations | % |
| --- | --- | --- | --- | --- |
| Hospital | 112,204 | 77.5% | 129,363 | 70.5% |
| Hospice | 2,249 | 1.6% | 3,068 | 1.7% |
| Care home | 22,546 | 15.6% | 37,768 | 20.6% |
| Home | 7,074 | 4.9% | 12,106 | 6.6% |
| Other | 793 | 0.5% | 1,289 | 0.7% |

Table S4: Distribution of COVID-19 deaths reported by UK Health Security Agency (UKHSA) and Office for National Statistics (ONS), by UKHSA region

| UKHSA Region | UKHSA 28-day measure | % | ONS COVID-19 death registrations | % |
| --- | --- | --- | --- | --- |
| East Midlands | 16,215 | 9.3% | 16,855 | 9.2% |
| East of England | 21,009 | 12.1% | 21,630 | 11.8% |
| London | 21,958 | 12.6% | 25,257 | 13.8% |
| North East | 9,752 | 5.6% | 9,982 | 5.4% |
| North West | 27,807 | 16.0% | 28,440 | 15.5% |
| South East | 25,211 | 14.5% | 27,728 | 15.1% |
| South West | 13,048 | 7.5% | 13,241 | 7.2% |
| West Midlands | 20,786 | 12.0% | 21,601 | 11.8% |
| Yorkshire and Humber | 18,006 | 10.4% | 18,742 | 10.2% |

Table S5: Distribution of COVID-19 deaths reported by UK Health Security Agency (UKHSA) and Office for National Statistics (ONS), by Index of Multiple Deprivation

| Index of Multiple Deprivation | UKHSA 28-day measure | % | ONS COVID-19 death registrations | % |
| --- | --- | --- | --- | --- |
| 1 (most deprived) | 40,493 | 23.3% | 42,376 | 23.1% |
| 2 | 36,835 | 21.2% | 38,812 | 21.2% |
| 3 | 34,392 | 19.8% | 36,265 | 19.8% |
| 4 | 32,688 | 18.8% | 34,642 | 18.9% |
| 5 (least deprived) | 29,384 | 16.9% | 31,381 | 17.1% |
